# Periodical Headache

**Published:** 1868-04-01

**Authors:** 


### *Periodical Headache.*

Dr. L. G. A., in common with many others, wishes *more light* on the subject of periodical headaches, as connected with menstruation — one of the most annoying tribulations to which females in this latitude and longitude are subjected. The book writers say little about it, and practically seem to know less. We should like at least a dozen communications, of a practical character, on the subject.

